# Supplementary material for: Genes Involved by Dexamethasone in Prevention of Long-Term Memory Impairment Caused by Lipopolysaccharide-Induced Neuroinflammation
Source: Biomedicines. 2023 Sep 22;11(10):2595. doi: 10.3390/biomedicines11102595 (PMC10604440; doi:10.3390/biomedicines11102595)
Supplement: Supplementary file 1 [file biomedicines-11-02595-s001.zip › biomedicines-2558353-supplementary.pdf]

Supplementary Materials: Additional Materials 1,2

Supplementary Materials: Additional Materials 1: The full original images of western blots with legends

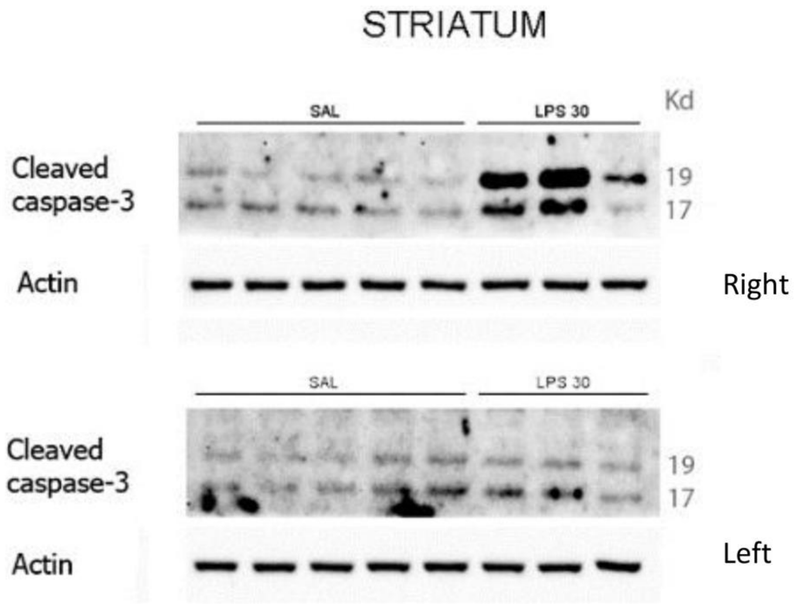

Pro-inflammatory-active caspase-3 P19 presented on immunoblot on the Figure 1 (b) in the manuscript, and both forms presented on the original blots here.

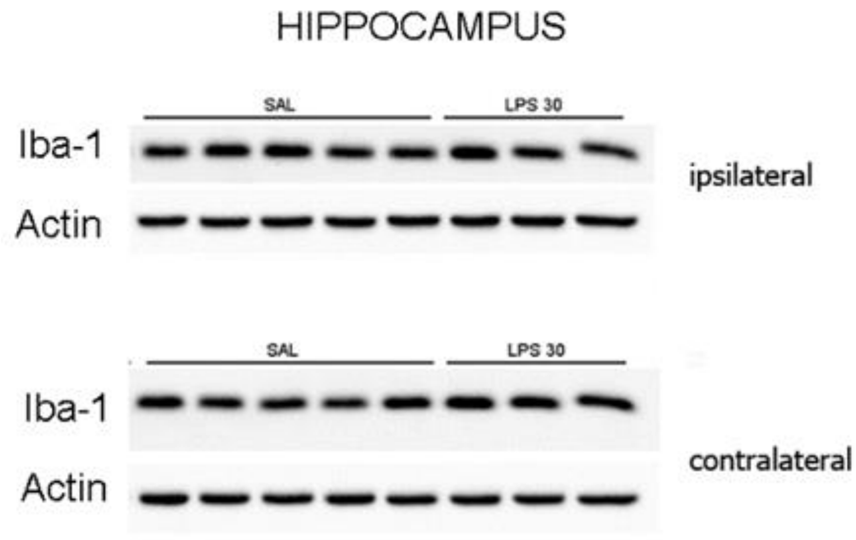

Supplementary Materials: Additional Materials 2: Representative IHC images of the right and left striatum.

Right striatum

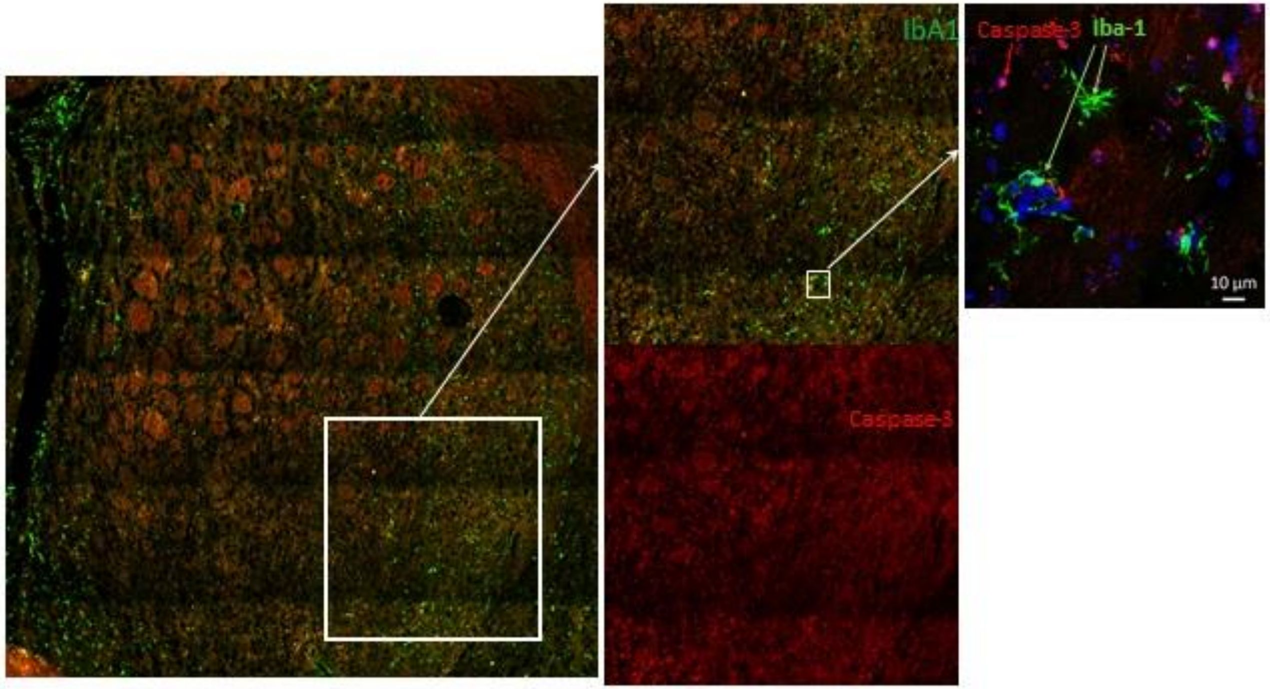

Left striatum

In contrast to the right striatum, no cells expressing active caspase-3 were found in the left striatum.

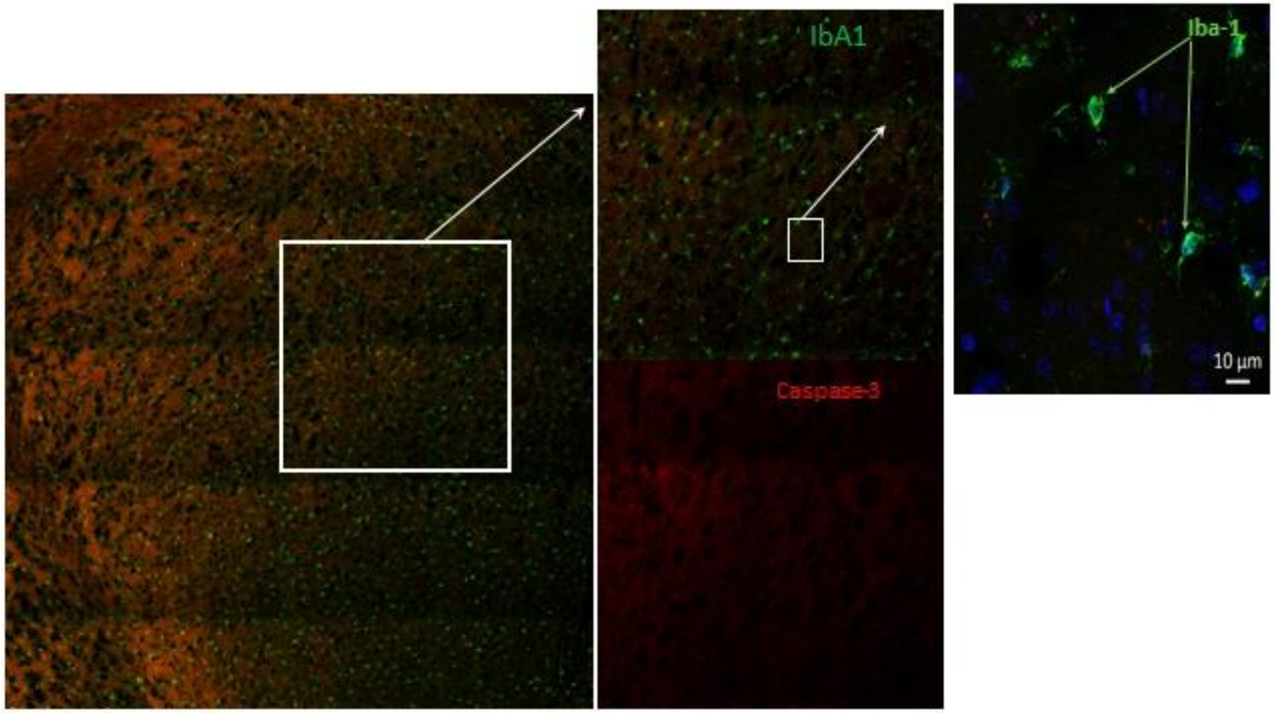

Supplementary Materials: Tables 1-5

Supplementary Materials, Table 1: List of differentially expressed genes with padj <0.05 and │log2FC│ ≥ 1 in the hippocampus of LPS vs. SAL

	Ensembl gene ID	Pos	Gene symbol	log2FC	pvalue	padj
1.	ENSRNOG00000000768	20:1876173-1897814	Ubd	6.58	2.62E-08	2.16E-05
2.	ENSRNOG00000024899	14:15253125-15258207	Cxcl13	5.84	2.07E-17	2.94E-14
3.	ENSRNOG00000032708	20:4039413-4049711	RT1-Bb	5.7	4.94E-13	5.53E-10
4.	ENSRNOG00000033215	20:4087618-4097190	RT1-Db1	4.48	1.07E-23	2.41E-20
5.	ENSRNOG00000032844	20:4127644-4132616	RT1-Da	4.4	7.29E-29	3.81E-25
6.	ENSRNOG00000000451	20:4066132-4070721	RT1-Ba	4.19	6.37E-23	1.25E-19
7.	ENSRNOG00000018735	18:56071478-56080849	Cd74	3.86	2.45E-27	7.67E-24
8.	ENSRNOG00000004649	3:121876263-121882726	Il1b	3.5	1.84E-10	1.80E-07
9.	ENSRNOG00000030431	20:4106189-4125387	RT1-Db2	3.41	4.28E-05	1.18E-02
10.	ENSRNOG00000042220	3:146484238-146491837	Vsx1	3.29	7.59E-07	4.10E-04
11.	ENSRNOG00000002217	14:10692764-10714524	Plac8	3.15	4.12E-06	1.70E-03
12.	ENSRNOG00000046834	9:9721105-9747167	C3	3.02	6.08E-22	1.06E-18
13.	ENSRNOG00000037167	8:119260618-119265157	Rtp3	2.89	2.36E-04	4.20E-02
14.	ENSRNOG00000033984	5:154037202-154058880	Ifnlr1	2.68	1.23E-13	1.49E-10
15.	ENSRNOG00000014464	16:85275678-85306366	Tnfsf13b	2.56	8.42E-08	5.89E-05
16.	ENSRNOG00000002659	10:5213350-5260608	Ciita	2.27	5.36E-07	3.00E-04
17.	ENSRNOG00000016294	4:157383052-157408176	Cd4	2.15	5.25E-24	1.37E-20
18.	ENSRNOG00000012789	19:55176258-55183557	Mlnr	2.11	5.03E-06	2.02E-03
19.	ENSRNOG00000033444	16:81803110-81822716	F10	2.03	9.87E-07	5.16E-04
20.	ENSRNOG00000014227	1:103298174-103323476	Mrgprx3	1.87	1.84E-07	1.11E-04
21.	ENSRNOG00000024000	1:89314558-89329418	Cd22	1.79	3.19E-09	2.94E-06
22.	ENSRNOG00000008134	10:12046541-12056311	Mefv	1.55	2.16E-04	3.94E-02
23.	ENSRNOG00000046254	9:9431860-9585865	Adgre1	1.5	1.56E-16	2.04E-13
24.	ENSRNOG00000001480	12:25497104-25506300	Ncf1	1.32	8.65E-08	5.89E-05
25.	ENSRNOG00000017980	1:198744050-198781750	Itgal	1.19	8.47E-05	2.01E-02
26.	ENSRNOG00000000187	7:119554354-119568776	Csf2rb	1.07	1.81E-05	5.90E-03
27.	ENSRNOG00000001959	11:37891156-37914983	Mx1	1.02	2.43E-04	4.27E-02
28.	ENSRNOG00000001216	20:11436267-11482051	Trpm2	0.95	7.74E-12	8.09E-09
29.	ENSRNOG00000007350	7:119783849-119797098	Rac2	0.95	5.56E-05	1.40E-02

30.	ENSRNOG00000012972	4:148398892-148446303	Alox5	-0.96	1.43E-04	2.76E-02
31.	ENSRNOG00000031207	4:40161285-40169564	LOC500035	-1.04	2.33E-27	7.67E-24
32.	ENSRNOG00000002979	13:83403264-83425641	Tbx19	-1.06	1.93E-04	3.60E-02
33.	ENSRNOG00000047459	17:43734461-43735120	H1f4	-1.98	1.25E-05	4.35E-03

Supplementary Materials, Table 2: List of differentially expressed genes with padj <0.05 and │log2FC│ ≥ 1 in the hippocampus of DEX+LPS vs. DEX+SAL

	Ensembl gene ID	Pos	Gene symbol	log2FC	pvalue	padj
1.	ENSRNOG00000024899	14:15253125-15258207	Cxcl13	7.4	3.61E-12	3.62E-10
2.	ENSRNOG00000032708	20:4039413-4049711	RT1-Bb	6.4	2.48E-25	9.25E-23
3.	ENSRNOG00000000451	20:4066132-4070721	RT1-Ba	5.7	4.30E-19	7.85E-17
4.	ENSRNOG00000033215	20:4087618-4097190	RT1-Db1	5.6	3.50E-20	7.24E-18
5.	ENSRNOG00000032844	20:4127644-4132616	RT1-Da	5.6	4.96E-18	7.84E-16
6.	ENSRNOG00000018735	18:56071478-56080849	Cd74	5.3	1.52E-15	1.96E-13
7.	ENSRNOG00000026762	20:3889370-3892449	RT1-DOa	4.9	1.65E-05	5.30E-04
8.	ENSRNOG00000000768	20:1876173-1897814	Ubd	4.1	3.30E-06	1.29E-04
9.	ENSRNOG00000037167	8:119260618-119265157	Rtp3	3.7	7.13E-06	2.52E-04
10.	ENSRNOG00000004649	3:121876263-121882726	Il1b	3.6	1.32E-10	1.13E-08
11.	ENSRNOG00000046834	9:9721105-9747167	C3	3.2	7.86E-23	2.23E-20
12.	ENSRNOG00000002659	10:5213350-5260608	Ciita	3.2	1.87E-08	1.11E-06
13.	ENSRNOG00000017485	5:58943027-58950373	Cd72	3.2	1.47E-05	4.78E-04
14.	ENSRNOG00000001724	11:73936750-73963852	Atp13a3	2.7	1.33E-07	6.81E-06
15.	ENSRNOG00000004947	7:15024693-15027933	Zfp871	2.7	1.55E-25	6.09E-23
16.	ENSRNOG00000016921	10:88378751-88389347	Klhl11	2.6	3.14E-55	8.01E-52
17.	ENSRNOG00000033984	5:154037202-154058880	Ifnlr1	2.6	1.33E-08	8.45E-07
18.	ENSRNOG00000006569	6:147094097-147172813	Itgb8	2.6	9.93E-32	5.44E-29
19.	ENSRNOG00000010996	14:20960052-20992753	Mob1b	2.5	1.74E-24	5.80E-22
20.	ENSRNOG00000042220	3:146484238-146491837	Vsx1	2.5	8.03E-04	1.47E-02
21.	ENSRNOG00000014227	1:103298174-103323476	Mrgprx3	2.4	2.77E-11	2.53E-09
22.	ENSRNOG00000010699	8:96395317-96401465	Trim43a	2.4	2.90E-04	6.12E-03
23.	ENSRNOG00000014464	16:85275678-85306366	Tnfsf13b	2.3	1.98E-06	8.08E-05
24.	ENSRNOG00000002537	X:20316893-20454357	Wnk3	2.3	1.01E-22	2.81E-20
25.	ENSRNOG00000011623	2:41570597-41785792	Rab3c	2.3	5.97E-62	3.05E-58
26.	ENSRNOG00000006967	X:128409472-128453000	Xiap	2.3	2.08E-22	5.69E-20

27.	ENSRNOG00000014511	7:131330913-131336183	Alg10	2.3	6.28E-22	1.66E-19
28.	ENSRNOG00000011341	2:56052643-56101803	Rictor	2.1	3.24E-38	2.48E-35
29.	ENSRNOG00000021552	X:75382598-75407217	Uprt	2.1	1.08E-10	9.37E-09
30.	ENSRNOG00000004828	3:44270653-44342355	Acvr1c	2.1	3.04E-23	9.14E-21
31.	ENSRNOG00000016294	4:157383052-157408176	Cd4	2.1	1.59E-07	8.05E-06
32.	ENSRNOG00000006583	4:95946025-95970666	Hpgds	2.1	1.28E-05	4.25E-04
33.	ENSRNOG00000002496	11:66316606-66566331	Stxbp5l	2	2.86E-73	4.39E-69
34.	ENSRNOG00000014891	1:241501679-241557651	Ptar1	2	5.31E-21	1.18E-18
35.	ENSRNOG00000026907	13:78836587-78852182	Zbtb37	2	7.78E-07	3.47E-05
36.	ENSRNOG00000000454	20:4020317-4026346	RT1-DOb	2	5.50E-05	1.50E-03
37.	ENSRNOG00000003748	X:54390733-54409466	RGD1565785	2	5.44E-04	1.05E-02
38.	ENSRNOG00000003680	10:27973681-28187565	Gabrb2	2	7.28E-41	8.58E-38
39.	ENSRNOG00000010065	15:60803098-60959769	Dgkh	1.9	5.26E-24	1.71E-21
40.	ENSRNOG00000047211	15:48601266-48670257	Fzd3	1.9	1.83E-42	2.55E-39
41.	ENSRNOG00000007706	5:124574079-124642569	Prkaa2	1.9	1.78E-34	1.18E-31
42.	ENSRNOG00000037919	X:74200972-74244846	Chic1	1.8	9.62E-11	8.42E-09
43.	ENSRNOG00000046515	9:6966908-7053387	St6gal2	1.8	2.02E-10	1.69E-08
44.	ENSRNOG00000038190	18:86420361-86878142	Dok6	1.8	2.06E-43	3.52E-40
45.	ENSRNOG00000012176	18:68488942-68551558	Rab27b	1.8	1.02E-21	2.60E-19
46.	ENSRNOG00000046254	9:9431860-9585865	Adgre1	1.8	3.47E-10	2.85E-08
47.	ENSRNOG00000014089	18:24961245-25025897	Map3k2	1.7	2.14E-11	1.99E-09
48.	ENSRNOG00000013624	1:102982123-103014388	Uevld	1.7	3.73E-10	3.00E-08
49.	ENSRNOG00000014248	9:75021790-75528644	Erbb4	1.7	2.65E-19	4.90E-17
50.	ENSRNOG00000014486	1:245859633-246110218	Rfx3	1.7	5.11E-30	2.45E-27
51.	ENSRNOG00000005479	3:92640752-92665644	Slc1a2	1.7	1.19E-31	6.06E-29
52.	ENSRNOG00000005206	7:106717229-107009330	Kcnq3	1.7	6.01E-61	2.30E-57
53.	ENSRNOG00000027152	14:44038614-44078897	N4bp2	1.7	4.45E-12	4.34E-10
54.	ENSRNOG00000010484	5:100924622-100977902	Zdhhc21	1.7	3.61E-26	1.45E-23
55.	ENSRNOG00000033942	10:5930298-6119990	Grin2a	1.7	1.98E-57	6.06E-54
56.	ENSRNOG00000005359	3:51883559-52120290	Csrnp3	1.7	1.95E-19	3.69E-17
57.	ENSRNOG00000009542	6:97872831-98157087	Kcnh5	1.6	7.40E-05	1.93E-03
58.	ENSRNOG00000001480	12:25497104-25506300	Ncf1	1.6	1.58E-18	2.63E-16
59.	ENSRNOG00000031669	11:79205730-79703736	Lpp	1.6	9.57E-04	1.70E-02

60.	ENSRNOG00000014550	2:54191538-54360780	Plcxd3	1.6	3.19E-05	9.34E-04
61.	ENSRNOG00000017980	1:198744050-198781750	Itgal	1.6	1.41E-07	7.22E-06
62.	ENSRNOG00000011059	3:112684152-112789289	Ttbk2	1.6	8.91E-29	4.01E-26
63.	ENSRNOG00000027151	11:65747036-65759581	Lrrc58	1.6	5.97E-23	1.76E-20
64.	ENSRNOG00000049849	X:107695491-107723594	Fam199x	1.6	1.57E-04	3.70E-03
65.	ENSRNOG00000025160	2:224772893-224802997	Tlcd4	1.6	1.88E-17	2.88E-15
66.	ENSRNOG00000005578	8:32165810-32217476	Zbtb44	1.5	2.88E-18	4.65E-16
67.	ENSRNOG00000025539	1:235653121-235813030	Vps13a	1.5	4.93E-22	1.33E-19
68.	ENSRNOG00000018268	19:31524671-31614246	Hhip	1.5	6.41E-14	7.39E-12
69.	ENSRNOG00000003434	13:60546912-60567882	Ro60	1.5	9.80E-09	6.37E-07
70.	ENSRNOG00000011858	16:67350539-67595225	Unc5d	1.4	5.66E-43	8.67E-40
71.	ENSRNOG00000011460	1:13838707-13915594	Arfgef3	1.4	6.92E-38	5.05E-35
72.	ENSRNOG00000008846	5:16791523-16799776	Plag1	1.4	1.18E-18	2.03E-16
73.	ENSRNOG00000000414	20:34575950-34684418	Cep85l	1.4	5.48E-14	6.36E-12
74.	ENSRNOG00000033531	4:15710417-16130848	Cacna2d1	1.4	2.49E-34	1.59E-31
75.	ENSRNOG00000013388	9:61066175-61134963	Pgap1	1.4	9.58E-20	1.88E-17
76.	ENSRNOG00000034190	6:138054662-138662511	Ighm	1.4	5.62E-05	1.52E-03
77.	ENSRNOG00000016334	5:76673990-76756147	Ptbp3	1.4	6.64E-18	1.04E-15
78.	ENSRNOG00000002790	X:75150608-75291938	Abcb7	1.4	2.41E-12	2.48E-10
79.	ENSRNOG00000005159	7:112673465-112833083	Fam135b	1.4	2.57E-12	2.62E-10
80.	ENSRNOG00000004150	X:2435305-2614770	Slc9a7	1.3	7.27E-28	3.18E-25
81.	ENSRNOG00000007528	3:48671079-48831467	Kcnh7	1.3	2.02E-14	2.41E-12
82.	ENSRNOG00000011696	2:56426367-56489415	Lifr	1.3	1.73E-31	8.53E-29
83.	ENSRNOG00000032206	13:21678512-22590586	Cntnap5b	1.3	9.93E-09	6.42E-07
84.	ENSRNOG00000016429	1:151439409-151783392	Grm5	1.3	5.41E-40	4.60E-37
85.	ENSRNOG00000027430	9:76621230-76768806	Ikzf2	1.3	3.70E-03	4.86E-02
86.	ENSRNOG00000001930	11:76747865-76804510	Ccdc50	1.3	5.21E-21	1.17E-18
87.	ENSRNOG00000009145	15:84324470-84748525	Klf12	1.3	1.70E-18	2.77E-16
88.	ENSRNOG00000024000	1:89314558-89329418	Cd22	1.3	8.18E-05	2.11E-03
89.	ENSRNOG00000005214	14:100151210-100217913	Plek	1.2	4.28E-12	4.20E-10
90.	ENSRNOG00000026306	4:68808359-68819928	Clec5a	1.2	1.98E-05	6.17E-04
91.	ENSRNOG00000026962	7:53878610-54027892	Osbpl8	1.2	5.98E-25	2.08E-22
92.	ENSRNOG00000011508	X:65040104-65074712	Zc3h12b	1.2	1.11E-18	1.93E-16

93.	ENSRNOG00000005639	X:67656253-67829026	Ar	1.2	4.94E-14	5.78E-12
94.	ENSRNOG00000048161	8:114916122-114920171	Tlr9	1.2	1.22E-06	5.26E-05
95.	ENSRNOG00000003742	X:35599258-35771711	Cdkl5	1.2	1.79E-21	4.50E-19
96.	ENSRNOG00000009899	2:115866701-115891097	Skil	1.2	1.57E-40	1.72E-37
97.	ENSRNOG00000006515	X:63343546-63389989	Klhl15	1.2	8.43E-04	1.53E-02
98.	ENSRNOG00000045771	4:70252366-70330803	Chl1	1.2	1.87E-27	7.97E-25
99.	ENSRNOG00000046452	13:89327794-89433815	Fcgr2b	1.2	1.32E-03	2.19E-02
100.	ENSRNOG00000022196	9:66568074-66676494	Bmpr2	1.1	2.01E-17	3.05E-15
101.	ENSRNOG00000011358	3:94352969-94419048	Hipk3	1.1	2.70E-38	2.18E-35
102.	ENSRNOG00000004218	6:86684413-86713370	Klhl28	1.1	5.44E-05	1.49E-03
103.	ENSRNOG00000031495	17:22832641-22863966	Tmem170b	1.1	3.02E-16	4.06E-14
104.	ENSRNOG00000015692	10:62566732-62630155	Taok1	1.1	7.35E-25	2.50E-22
105.	ENSRNOG00000034269	5:144364269-144436509	Ago3	1.1	2.58E-10	2.13E-08
106.	ENSRNOG00000013886	2:55835151-55983804	Fyb1	1.1	1.21E-09	9.06E-08
107.	ENSRNOG00000008039	8:58202821-58253688	Cul5	1.1	7.74E-32	4.39E-29
108.	ENSRNOG00000011619	8:64573358-64777543	Myo9a	1.1	2.06E-29	9.57E-27
109.	ENSRNOG00000012367	14:55072200-55081551	Pcdh7	1.1	3.89E-17	5.79E-15
110.	ENSRNOG00000011063	13:56015901-56236677	Dennd1b	1.1	3.61E-07	1.71E-05
111.	ENSRNOG00000013281	18:1970914-2094920	Mib1	1.1	2.36E-42	3.02E-39
112.	ENSRNOG00000048686	9:8349033-8442298	AABR07066379.1	1.1	2.66E-05	7.99E-04
113.	ENSRNOG00000042679	1:260732485-260830854	Lcor	1.1	6.15E-15	7.66E-13
114.	ENSRNOG00000013412	9:71230108-71293435	Creb1	1.1	1.94E-04	4.40E-03
115.	ENSRNOG00000013884	16:23447366-23781604	Psd3	1	5.32E-40	4.60E-37
116.	ENSRNOG00000012759	9:30515089-30844199	Col19a1	1	3.52E-09	2.47E-07
117.	ENSRNOG00000029662	16:9194424-9430743	Wdfy4	1	2.26E-07	1.10E-05
118.	ENSRNOG00000037563	10:56268720-56270640	Cd68	1	1.90E-03	2.92E-02
119.	ENSRNOG00000005957	15:11836286-11912916	Slc4a7	1	1.41E-08	8.92E-07
120.	ENSRNOG00000009196	3:21623041-21670014	Rc3h2	1	2.17E-19	4.06E-17
121.	ENSRNOG00000006980	5:9230984-9257934	Vcpip1	1	1.38E-18	2.32E-16
122.	ENSRNOG00000025937	8:21448618-21458379	Zfp26	1	1.40E-13	1.58E-11
123.	ENSRNOG00000002863	13:71906702-72367980	Cacna1e	1	4.11E-36	2.86E-33
124.	ENSRNOG00000000277	20:27366213-27437427	Tet1	1	1.75E-05	5.56E-04
125.	ENSRNOG00000002075	14:15059387-15146586	Cnot6l	1	4.70E-21	1.11E-18

126. ENSRNOG00000004378	10:98576039-98644938	Abca5	1	1.85E-09	1.34E-07
127. ENSRNOG00000046969	11:71634392-71695000	AABR07034438.1	1	1.91E-06	7.81E-05
128. ENSRNOG00000008736	13:90967739-90977734	Slamf8	1	2.72E-03	3.83E-02
129. ENSRNOG00000022331	10:15156207-15159894	Ccdc78	-1	1.65E-05	5.31E-04
130. ENSRNOG00000022723	9:66335492-66447837	RGD1562029	-1	1.36E-04	3.28E-03
131. ENSRNOG00000014893	2:251912368-251970768	Wdr63	-1	4.23E-05	1.19E-03
132. ENSRNOG00000010529	1:56653938-56683731	Thbs2	-1	2.20E-03	3.28E-02
133. ENSRNOG00000021437	5:155812105-155885829	AABR07073181.1	-1	1.64E-08	1.01E-06
134. ENSRNOG00000012972	4:148398892-148446303	Alox5	-1	9.88E-06	3.36E-04
135. ENSRNOG00000003952	13:44523797-44540516	AABR07020879.1	-1	1.65E-05	5.31E-04
136. ENSRNOG00000029586	8:116776494-116779156	AC128059.1	-1	1.35E-04	3.26E-03
137. ENSRNOG00000023561	4:158224000-158576978	Ano2	-1	2.17E-03	3.23E-02
138. ENSRNOG00000024172	1:52907435-52962388	T2	-1	6.79E-05	1.80E-03
139. ENSRNOG00000007490	5:48303366-48341642	Gabrr2	-1.1	1.95E-03	2.97E-02
140. ENSRNOG00000025860	19:10363108-10380809	Drc7	-1.1	1.19E-05	3.99E-04
141. ENSRNOG00000047124	1:197856903-197858016	AABR07005775.1	-1.1	1.02E-03	1.79E-02
142. ENSRNOG00000047455	6:52345240-52401853	Cdhr3	-1.1	1.08E-03	1.87E-02
143. ENSRNOG00000026914	16:7345978-7408265	Dnah1	-1.2	1.18E-07	6.13E-06
144. ENSRNOG00000014937	2:187102833-187113752	Lrrc71	-1.2	1.71E-08	1.04E-06
145. ENSRNOG00000008911	5:164913906-164927869	Draxin	-1.2	4.34E-05	1.22E-03
146. ENSRNOG00000037668	20:46271689-46305157	LOC102554658	-1.3	7.76E-08	4.16E-06
147. ENSRNOG00000015101	17:21521505-21568440	Mak	-1.3	1.44E-03	2.33E-02
148. ENSRNOG00000016247	9:88964525-89001567	Daw1	-1.4	3.18E-05	9.34E-04
149. ENSRNOG00000018250	1:72882806-72886488	Tnni3	-1.4	1.66E-03	2.60E-02
150. ENSRNOG00000037206	4:152860675-152883210	Ccdc77	-1.4	2.00E-06	8.13E-05
151. ENSRNOG00000038600	1:72874404-72883002	Dnaaf3	-1.4	2.32E-06	9.32E-05
152. ENSRNOG00000012040	17:8570472-8607494	Slc25a48	-1.5	9.30E-04	1.66E-02
153. ENSRNOG00000012342	19:33206026-33424972	Ttc29	-1.5	9.82E-05	2.46E-03
154. ENSRNOG00000012229	1:52887067-52894832	Tbxt	-1.5	5.72E-04	1.10E-02
155. ENSRNOG00000020132	5:173471010-173484986	Ttll10	-1.6	3.56E-05	1.03E-03
156. ENSRNOG00000032085	8:127789048-127834637	Dlec1	-1.7	8.78E-10	6.73E-08
157. ENSRNOG00000027392	3:3724656-3747440	Ccdc187	-1.8	2.09E-05	6.44E-04
158. ENSRNOG00000015049	8:128169191-128266639	Scn5a	-1.8	7.91E-05	2.04E-03

159. ENSRNOG00000047459	17:43734461-43735120	H1f4	-1.8	4.77E-05	1.33E-03
160. ENSRNOG00000049495	7:143345201-143353925	Krt71	-1.9	1.23E-03	2.07E-02
161. ENSRNOG00000001193	20:10757854-10844178	Hsf2bp	-2.6	1.02E-04	2.55E-03

Supplementary Materials, Table 3: venn_result27926

Names	total	elements
LPS vs. SAL _ DEX+LPS vs. DEX+SAL	22	ENSRNOG00000046834
		ENSRNOG00000012972
		ENSRNOG00000014227
		ENSRNOG00000032708
		ENSRNOG00000042220
		ENSRNOG00000024899
		ENSRNOG00000000768
		ENSRNOG00000047459
		ENSRNOG00000033984
		ENSRNOG00000017980
		ENSRNOG00000002659
		ENSRNOG00000004649
		ENSRNOG00000033215
		ENSRNOG00000016294
		ENSRNOG00000032844
		ENSRNOG00000014464
		ENSRNOG00000037167
		ENSRNOG00000000451
		ENSRNOG00000001480
		ENSRNOG00000046254
		ENSRNOG00000024000
		ENSRNOG00000018735
DEX+LPS vs. DEX+SAL _ DEX+LPS vs. DEX	84	ENSRNOG00000004150
		ENSRNOG00000010699
		ENSRNOG00000004828
		ENSRNOG00000018268
		ENSRNOG00000005159

ENSRNOG00000033531
ENSRNOG00000007706
ENSRNOG00000000414
ENSRNOG00000011460
ENSRNOG00000025539
ENSRNOG00000003742
ENSRNOG00000009899
ENSRNOG00000014511
ENSRNOG00000033942
ENSRNOG00000011508
ENSRNOG00000006569
ENSRNOG00000001724
ENSRNOG00000014089
ENSRNOG00000010996
ENSRNOG00000002075
ENSRNOG00000005206
ENSRNOG00000037919
ENSRNOG00000009145
ENSRNOG00000009542
ENSRNOG00000006980
ENSRNOG00000003680
ENSRNOG00000031495
ENSRNOG00000016334
ENSRNOG00000006967
ENSRNOG00000002496
ENSRNOG00000026907
ENSRNOG00000027151
ENSRNOG00000013884
ENSRNOG00000011358
ENSRNOG00000013388
ENSRNOG00000016921
ENSRNOG00000004218
ENSRNOG00000013281

ENSRNOG00000021552
ENSRNOG00000045771
ENSRNOG00000011619
ENSRNOG00000025160
ENSRNOG00000026962
ENSRNOG00000034269
ENSRNOG00000004947
ENSRNOG00000005578
ENSRNOG00000012759
ENSRNOG00000049849
ENSRNOG00000011341
ENSRNOG00000046515
ENSRNOG00000011063
ENSRNOG00000038190
ENSRNOG00000042679
ENSRNOG00000011858
ENSRNOG00000011623
ENSRNOG00000000277
ENSRNOG00000014486
ENSRNOG00000002537
ENSRNOG00000001930
ENSRNOG00000013412
ENSRNOG00000005639
ENSRNOG00000010484
ENSRNOG00000002863
ENSRNOG00000011696
ENSRNOG00000047211
ENSRNOG00000010065
ENSRNOG00000005479
ENSRNOG00000015692
ENSRNOG00000009196
ENSRNOG00000027152
ENSRNOG00000022196

		ENSRNOG00000032206
		ENSRNOG00000008846
		ENSRNOG00000016429
		ENSRNOG00000014248
		ENSRNOG00000012176
		ENSRNOG00000046969
		ENSRNOG00000005359
		ENSRNOG00000003434
		ENSRNOG00000011059
		ENSRNOG00000025937
		ENSRNOG00000014891
		ENSRNOG00000013624
		ENSRNOG00000007528
LPS vs. SAL	11	ENSRNOG00000007350
		ENSRNOG00000001959
		ENSRNOG00000002217
		ENSRNOG00000031207
		ENSRNOG00000001216
		ENSRNOG00000030431
		ENSRNOG00000008134
		ENSRNOG00000002979
		ENSRNOG00000033444
		ENSRNOG00000012789
		ENSRNOG00000000187
DEX+LPS vs. DEX+SAL	55	ENSRNOG00000023561
		ENSRNOG00000014550
		ENSRNOG00000025860
		ENSRNOG00000008039
		ENSRNOG00000018250
		ENSRNOG00000029662
		ENSRNOG00000012229
		ENSRNOG00000031669
		ENSRNOG00000026306

ENSRNOG00000032085
ENSRNOG00000000454
ENSRNOG00000015101
ENSRNOG00000021437
ENSRNOG00000012342
ENSRNOG00000004378
ENSRNOG00000037563
ENSRNOG00000013886
ENSRNOG00000034190
ENSRNOG00000020132
ENSRNOG00000026762
ENSRNOG00000048686
ENSRNOG00000003952
ENSRNOG00000047455
ENSRNOG00000006583
ENSRNOG00000015049
ENSRNOG00000001193
ENSRNOG00000027392
ENSRNOG00000007490
ENSRNOG00000003748
ENSRNOG00000010529
ENSRNOG00000026914
ENSRNOG00000046452
ENSRNOG00000047124
ENSRNOG00000022723
ENSRNOG00000022331
ENSRNOG00000005957
ENSRNOG00000037668
ENSRNOG00000006515
ENSRNOG00000024172
ENSRNOG00000016247
ENSRNOG00000029586
ENSRNOG00000027430

		ENSRNOG00000049495
		ENSRNOG00000014937
		ENSRNOG00000017485
		ENSRNOG00000005214
		ENSRNOG00000038600
		ENSRNOG00000037206
		ENSRNOG00000002790
		ENSRNOG00000014893
		ENSRNOG00000012040
		ENSRNOG00000048161
		ENSRNOG00000012367
		ENSRNOG00000008911
		ENSRNOG00000008736
DEX+LPS vs. LPS	28	ENSRNOG00000008444
		ENSRNOG00000010859
		ENSRNOG00000002941
		ENSRNOG00000003472
		ENSRNOG00000028362
		ENSRNOG00000027233
		ENSRNOG00000004118
		ENSRNOG00000049829
		ENSRNOG00000048800
		ENSRNOG00000023549
		ENSRNOG00000002372
		ENSRNOG00000027564
		ENSRNOG00000033479
		ENSRNOG00000010597
		ENSRNOG00000001547
		ENSRNOG00000018191
		ENSRNOG00000032180
		ENSRNOG00000003643
		ENSRNOG00000003882
		ENSRNOG00000003787

ENSRNOG00000042951
ENSRNOG00000024998
ENSRNOG00000012470
ENSRNOG00000008382
ENSRNOG00000008869
ENSRNOG00000045622
ENSRNOG00000024595
ENSRNOG00000010111

Supplementary Materials, Table 4: Enriched GO biological process terms for LPS vs. SAL DEGs

	Term (Category: GOTERM_BP_DIRECT)	Count	%	PValue	Genes
1.	GO:0019886~antigen processing and presentation of exogenous peptide antigen via MHC class II	6	18.75	2.14E-10	Cd74, RT1-Ba, RT1-Bb, RT1-Da, RT1-Db1, RT1-Db2
2.	GO:0002504~antigen processing and presentation of peptide or polysaccharide antigen via MHC class II	5	15.63	3.35E-10	RT1-Ba, RT1-Bb, RT1-Da, RT1-Db1, RT1-Db2
3.	GO:0034341~response to interferon-gamma	6	18.75	2.37E-09	Cd74, Mefv, RT1-Ba, RT1-Db1, Ciita, Ubd
4.	GO:0002503~peptide antigen assembly with MHC class II protein complex	5	15.63	3.40E-09	RT1-Ba, RT1-Bb, RT1-Da, RT1-Db1, RT1-Db2
5.	GO:0050778~positive regulation of immune response	5	15.63	5.94E-08	RT1-Ba, RT1-Bb, RT1-Da, RT1-Db1, RT1-Db2
6.	GO:0050870~positive regulation of T cell activation	5	15.63	3.42E-07	RT1-Ba, RT1-Bb, RT1-Da, RT1-Db1, RT1-Db2
7.	GO:0002250~adaptive immune response	6	18.75	6.78E-07	RT1-Ba, RT1-Bb, RT1-Da, RT1-Db1, RT1-Db2,Adgre1
8.	GO:0006954~inflammatory response	7	21.88	1.62E-05	Cxcl13, Mefv, Alox5, Ciita, C3, Il1b, Ncf1
9.	GO:0019882~antigen processing and presentation	4	12.5	5.64E-05	Cd74, RT1-Ba, RT1-Bb, RT1-Db2
10.	GO:0006955~immune response	6	18.75	9.82E-05	Cd74, RT1-Bb, RT1-Db1, RT1-Db2, Tnfsf13b, Il1b
11.	GO:0090023~positive regulation of neutrophil chemotaxis	3	9.38	8.66E-04	Cd74, Rac2, Il1b
12.	GO:0016064~immunoglobulin mediated immune response	3	9.38	1.13E-03	Cd74, RT1-Da, Csf2rb
13.	GO:0006959~humoral immune response	3	9.38	1.35E-03	RT1-Bb, RT1-Db1, Alox5
14.	GO:0002469~myeloid dendritic cell antigen processing and presentation	2	6.25	3.12E-03	RT1-Da, RT1-Db1
15.	GO:0002491~antigen processing and presentation of endogenous peptide antigen via MHC class II	2	6.25	3.12E-03	RT1-Da, RT1-Db1
16.	GO:0043123~positive regulation of I-kappaB kinase/NF-kappaB signaling	4	12.5	3.35E-03	Cd74, RT1-Db1, Il1b, Ubd
17.	GO:0042130~negative regulation of T cell proliferation	3	9.38	3.44E-03	RT1-Ba, RT1-Bb, RT1-Db1
18.	GO:2000516~positive regulation of CD4-positive, alpha-beta T cell activation	2	6.25	6.22E-03	RT1-Da, RT1-Db1
19.	GO:0042102~positive regulation of T cell proliferation	3	9.38	6.40E-03	Tnfsf13b, Itgal, Il1b
20.	GO:0070374~positive regulation of ERK1 and ERK2 cascade	4	12.5	6.43E-03	Cd74, RT1-Db1, C3, Il1b
21.	GO:0048002~antigen processing and presentation of peptide antigen	2	6.25	7.77E-03	RT1-Ba, RT1-Bb
22.	GO:0045622~regulation of T-helper cell differentiation	2	6.25	7.77E-03	RT1-Da, RT1-Db1

23.	GO:0001934~positive regulation of protein phosphorylation	4	12.5	8.05E-03	Cd74, RT1-Db1, C3, Il1b
24.	GO:0043382~positive regulation of memory T cell differentiation	2	6.25	9.32E-03	RT1-Da, RT1-Db1
25.	GO:0032831~positive regulation of CD4-positive, CD25-positive, alpha-beta regulatory T cell differentiation	2	6.25	1.09E-02	RT1-Da, RT1-Db1
26.	GO:0050729~positive regulation of inflammatory response	3	9.38	1.16E-02	Cd74, Mefv, Il1b
27.	GO:0031394~positive regulation of prostaglandin biosynthetic process	2	6.25	1.55E-02	Cd74, Il1b
28.	GO:0050691~regulation of defense response to virus by host	2	6.25	1.55E-02	Ifnlr1, Il1b
29.	GO:0045657~positive regulation of monocyte differentiation	2	6.25	1.70E-02	Cd74, RT1-Db1
30.	GO:0006691~leukotriene metabolic process	2	6.25	2.01E-02	Alox5, Ncf1
31.	GO:0046598~positive regulation of viral entry into host cell	2	6.25	2.01E-02	Cd74, RT1-Db1
32.	GO:0019221~cytokine-mediated signaling pathway	3	9.38	2.06E-02	Csf2rb, Ifnlr1, Il1b
33.	GO:0045893~positive regulation of transcription, DNA-templated	5	15.63	2.71E-02	Cd74, RT1-Db1, Ciita, Il1b, Ncf1
34.	GO:0045087~innate immune response	4	12.5	2.90E-02	Mefv, Mx1, Ciita, C3
35.	GO:0007166~cell surface receptor signaling pathway	3	9.38	2.93E-02	Cxcl13, Adgre1, Itgal
36.	GO:0045582~positive regulation of T cell differentiation	2	6.25	3.83E-02	Cd74, RT1-Ba
37.	GO:0042742~defense response to bacterium	3	9.38	3.83E-02	Cxcl13, Ncf1, Plac8
38.	GO:0002526~acute inflammatory response	2	6.25	4.13E-02	Alox5, Il1b
39.	GO:0010575~positive regulation of vascular endothelial growth factor production	2	6.25	4.28E-02	C3, Il1b
40.	GO:0046597~negative regulation of viral entry into host cell	2	6.25	4.73E-02	Cd74, Ciita
41.	GO:0050832~defense response to fungus	2	6.25	4.88E-02	RT1-Bb, Ncf1

Supplementary Materials, Table 5: Enriched GO biological process terms for DEX+LPS vs. DEX+SAL DEGs

	Term (Category: GOTERM_BP_DIRECT)	Count	%	PValue	Genes
1.	GO:0019886~antigen processing and presentation of exogenous peptide antigen via MHC class II	8	4,79	1,69E-10	Cd74, Fcgr2b, RT1-Ba, RT1-Bb, RT1-DOa, RT1-DOb, RT1-Da, RT1-Db1
2.	GO:0002381~immunoglobulin production involved in immunoglobulin mediated immune response	6	3,59	9,47E-09	RT1-Ba, RT1-Bb, RT1-DOa, RT1-DOb, RT1-Da, RT1-Db1
3.	GO:0002503~peptide antigen assembly with MHC class II protein complex	6	3,59	2,61E-08	RT1-Ba, RT1-Bb, RT1-DOa, RT1-DOb, RT1-Da, RT1-Db1
4.	GO:0002504~antigen processing and presentation of peptide or polysaccharide antigen via MHC class II	5	2,99	2,01E-07	RT1-Ba, RT1-Bb, RT1-DOb, RT1-Da, RT1-Db1
5.	GO:0050870~positive regulation of T cell activation	6	3,59	7,65E-06	RT1-Ba, RT1-Bb, RT1-DOa, RT1-DOb, RT1-Da, RT1-Db1
6.	GO:0019882~antigen processing and presentation	6	3,59	2,28E-05	Cd74, Rab3c, RT1-Ba, RT1-Bb, RT1-DOa, Wdfy4
7.	GO:0034341~response to interferon-gamma	5	2,99	1,44E-04	Cd74, RT1-Ba, RT1-Db1, Ciita, Ubd
8.	GO:0002250~adaptive immune response	7	4,19	1,49E-04	RT1-Ba, RT1-Bb, RT1-DOa, RT1-DOb, RT1-Da, RT1-Db1, Adgre1
9.	GO:0034765~regulation of ion transmembrane transport	6	3,59	1,28E-03	Cacna2d1, Cacna1e, Kcnh5, Kcnh7, Kcnq3, Scn5a
10.	GO:1901224~positive regulation of NIK/NF-kappaB signaling	5	2,99	1,84E-03	Cd74, Ago3, Il1b, Rc3h2, Tlr9
11.	GO:0050852~T cell receptor signaling pathway	5	2,99	5,97E-03	Dennd1b, Fyb1, RT1-Bb, RT1-Db1, Rc3h2

12.	GO:0046330~positive regulation of JNK cascade	5	2,99	6,87E-03	Fcgr2b, Taok1, Xiap, Il1b, Ncf1
13.	GO:0050776~regulation of immune response	4	2,39	7,58E-03	Cd22, Dennd1b, Fcgr2b, Tnfsf13b
14.	GO:0006955~immune response	8	4,79	7,65E-03	Cd74, Fyb1, RT1-Bb, RT1-Db1, Tnfsf13b, Itgb8, Il1b, Tlr9
15.	GO:0071363~cellular response to growth factor stimulus	5	2,99	8,13E-03	Acvr1c, Bmpr2, Creb1, Grin2a, Slc4a7
16.	GO:0006468~protein phosphorylation	11	6,58	1,14E-02	Taok1, Wnk3, Acvr1c, Bmpr2, Creb1, Cdkl5, Grm5, Hipk3, Mak, Map3k2. Prkaa2
17.	GO:0042391~regulation of membrane potential	5	2,99	1,24E-02	Gabrb2, Gabrr2, Grin2a, Kcnh5, Kcnh7
18.	GO:0002469~myeloid dendritic cell antigen processing and presentation	2	1,19	1,48E-02	RT1-Da, RT1-Db1
19.	GO:0002587~negative regulation of antigen processing and presentation of peptide antigen via MHC class II	2	1,19	1,48E-02	RT1-DOa, RT1-DOb
20.	GO:0002491~antigen processing and presentation of endogenous peptide antigen via MHC class II	2	1,19	1,48E-02	RT1-Da, RT1-Db1
21.	GO:0043410~positive regulation of MAPK cascade	6	3,59	1,80E-02	Cd74, RT1-Db1, Ar, Dok6, Grm5, Tlr9
22.	GO:0051649~establishment of localization in cell	6	3,59	1,80E-02	Fcgr2b, Ano2, Cacna1e, Daw1, Kcnq3, Scn5a
23.	GO:0048169~regulation of long-term neuronal synaptic plasticity	3	1,79	2,09E-02	Fcgr2b, Grin2a, Grm5
24.	GO:0086048~membrane depolarization during bundle of His cell action potential	2	1,19	2,22E-02	Cacna2d1, Scn5a
25.	GO:0016064~immunoglobulin mediated immune response	3	1,79	2,22E-02	Cd74, Fcgr2b, RT1-Da
26.	GO:0021549~cerebellum development	4	2,39	2,64E-02	Fcgr2b, Acvr1c, Scn5a)
27.	GO:0021537~telencephalon development	3	1,79	2,64E-02	Erbb4, Scn5a,
28.	GO:0006959~humoral immune response	3	1,79	2,79E-02	RT1-Bb, RT1-Db1, Alox5
29.	GO:2000516~positive regulation of CD4-positive, alpha-beta T cell activation	2	1,19	2,94E-02	RT1-Da, RT1-Db1
30.	GO:0071805~potassium ion transmembrane transport	5	2,99	2,95E-02	Alg10, Kcnh5, Kcnh7, Kcnq3, Slc9a7
31.	GO:0017157~regulation of exocytosis	3	1,79	3,09E-02	Rab27b, Rab3c, Stxbp5l
32.	GO:0035556~intracellular signal transduction	8	4,79	3,58E-02	Taok1, Wnk3, Dgkh, Mak, Map3k2, Myo9a, Plek
33.	GO:0048002~antigen processing and presentation of peptide antigen	2	1,19	3,67E-02	RT1-Ba, RT1-Bb
34.	GO:0045622~regulation of T-helper cell differentiation	2	1,19	3,67E-02	RT1-Da, RT1-Db1
35.	GO:0007166~cell surface receptor signaling pathway	5	2,99	3,70E-02	Cxcl13, Fcgr2b, Adgre1, Erbb4, Itgal
36.	GO:0050796~regulation of insulin secretion	3	1,79	3,90E-02	Alox5, Il1b, Rfx3
37.	GO:0006816~calcium ion transport	4	2,39	3,96E-02	Atp2a1, Cacna2d1, Cacna1e, Grin2a
38.	GO:0007229~integrin-mediated signaling pathway	4	2,39	4,06E-02	Fyb1, Itgal, Itgb8, Plek
39.	GO:0043382~positive regulation of memory T cell differentiation	2	1,19	4,38E-02	RT1-Da, RT1-Db1
40.	GO:0006612~protein targeting to membrane	3	1,79	4,60E-02	Ncf1, Rtp3, Zdhhc21
41.	GO:0050871~positive regulation of B cell activation	3	1,79	4,78E-02	Ighm, Ighm, Tlr9
42.	GO:0009410~response to xenobiotic stimulus	9	5,38	4,81E-02	Acvr1c, Creb1, C3, Erbb4, Fzd3, Grin2a, Hspg2, Il1b, Slc1a2
43.	GO:0006954~inflammatory response	7	4,19	4,92E-02	Cxcl13, Alox5, Ciita, C3, Il1b, Ncf1, Tlr9)

44. GO:0060078~regulation of postsynaptic membrane potential	3	1,79	4,96E-02	Gabrr2, Grin2a, Grm5
